# Relationship between body adiposity and glycemic control in children and adolescents with type 1 diabetes

**DOI:** 10.1007/s00592-026-02643-2

**Published:** 2026-01-31

**Authors:** Claudio Maffeis, Ilaria Fierri, Elisa Morotti, Erika Caiazza, Quincy Pedranzini, Marco Marigliano, Claudia Piona

**Affiliations:** Section of Pediatric Diabetes and Metabolism, Department of Surgery, Dentistry, Pediatrics, and Gynecology, University and Azienda Ospedaliera Universitaria Integrata of Verona, Piazzale Stefani, 1, Verona, 37126 Italy

**Keywords:** Type 1 diabetes, Children, Body mass index, Adiposity, HbA1c, TIR

## Abstract

**Aims:**

To investigate the relationship between body adiposity and glycemic control in children and adolescents with type 1 diabetes (T1D).

**Methods:**

This cross-sectional study included 364 children and adolescents aged 6–18 years with T1D. Anthropometric indices [BMI, BMI Z-score, waist-to-height ratio (WHtR)] and body composition [fat mass (FM), FM%, fat mass index (FMI)], assessed using bioelectrical impedance analysis, were obtained. Hemoglobin A1c and glucose sensor metrics, including time in range (TIR), were used to assess glycemic control. Associations between variables were analyzed using Spearman’s correlation. Logistic regression models were run to identify independent predictors of HbA1c < 7.0% and TIR > 70%, with FMI, WHtR, total daily insulin dose per kg (TDD), treatment modalities, sex, age, diabetes duration, and pubertal stage as independent variables.

**Results:**

Adiposity measures (FMI, FM%, and WHtR) were positively associated with HbA1c and negatively with TIR in both sexes. Logistic regression showed that HbA1c < 7% and TIR > 70% were significantly predicted by FMI [OR(95%CI): 0.822(0.704–0.960), *p* = 0.013, and 0.807(0.681–0.955), *p* = 0.012, respectively] and WHtR(x100) [OR(95%CI): 0.927(0.874–0.983), *p* = 0.013, and 0.923(0.866–0.985), *p* = 0.015, respectively], independently of TDD, *sex*, treatment modalities and the other independent variables.

**Conclusions:**

Body adiposity negatively impacts glycemic control in children and adolescents with T1D, independent of *sex and* insulin treatment modalities. Despite technological advances in diabetes care, excess adiposity is emerging as a key modifiable factor associated with poorer glycemic outcomes and, *consequently*,* poorer* long-term health in children and adolescents with T1D.

**Supplementary Information:**

The online version contains supplementary material available at 10.1007/s00592-026-02643-2.

## Introduction

Setting glycemic targets for young people with type 1 diabetes (PwD) is crucial for reducing both acute and long-term diabetes complications. This is particularly relevant for children and adolescents, who often present with early-onset type 1 diabetes (T1D). Recent consensus statements and guidelines from the International Society for Pediatric and Adolescent Diabetes (ISPAD) and the American Diabetes Association (ADA) recommended HbA1c < 7.0% or < 53 mmol/mol and time in range (TIR) > 70% as targets of optimal glycemic control for children and adolescents with T1D [1, 2]. However, the introduction of continuous glucose monitoring (CGM) and standard insulin pump (IP) therapy, followed by automated insulin delivery (AID) systems, has contributed to further improvements in glycemic control in PwD, leading to the proposal of more ambitious glycemic targets, such as HbA1c < 6.5% or < 48 mmol/mol [1, 2]. The use of AID systems has rapidly become the standard of care, allowing PwD to reach more stringent glycemic targets [3].

The continuous improvement in glycemic control, as reflected by these updated glycemic targets, is expected to reduce the risk of and delay the onset of micro- and macrovascular complications of T1D, which are still higher in PwD than in the general population [4, 5]. An improvement in survival among PwD has been recently observed: individuals over 45 years without prior renal or cardiovascular complications had standardized mortality rates like or even lower than those of controls [6].

Despite technology advancements in diabetes care, most children and adolescents with T1D still struggle to achieve glycemic targets, regardless of the treatment modalities, whether MDI, IP, or advanced AID systems [7, 8]. Several factors contribute to this challenge, including age, sex, diabetes duration, socioeconomic status (SES), unhealthy dietary habits, and psychosocial determinants, such as diabetes distress, family dynamics, mental health, and adherence to therapy [9–11].

Another potential contributing factor to suboptimal glucose control is excess body weight. Indeed, the prevalence of overweight and obesity is continuously increasing worldwide [12] and is described in almost 30% of children and adolescents with T1D [13–15]. This trend results from the complex interplay between an obesogenic environment, including unhealthy eating behaviors, and genetic predisposition, both of which promote fat accumulation and weight gain [16, 17]. Excess body weight is increasingly recognized as an independent factor negatively affecting PwD, determining a higher risk of diabetes complications [18]. A significant association was found between lipid profiles, blood pressure, and body composition in children and adolescents with T1D, with higher BMI and adiposity linked to increased exposure to hypertension, cardiovascular risk factors (CVRFs), and insulin resistance [19]. Some studies suggest that excess body weight has a direct impact on glycemic control, as adolescents with a higher BMI are less likely to meet international glycemic targets than their normal-weight peers [20, 21]. In addition, the use of IP and AID can lead to an increase in excess body weight, as indicated by higher BMI Z-scores, among youth using diabetes technology, and to overall increasing trends in BMI following the initiation of IP [20, 22]. These studies primarily assessed excess body weight using the BMI as a rough indicator of adiposity, rather than more direct measures of body adiposity. Moreover, they did not comprehensively evaluate CGM metrics and the most recent treatment modalities for T1D. Thus, this study examines the relationship between glycemic control and body fat mass in children and adolescents with T1D, investigating how various treatment modalities affect this association. The aim was to generate a potential hypothesis regarding the independent role of body adiposity in glycemic control among children and adolescents with T1D.

## Materials and methods

### Study protocol and participants

Three hundred sixty-four children and adolescents with T1D were recruited at the Regional Center for Pediatric Diabetes of Verona, Italy. Inclusion criteria: age (6–18 years), confirmed diagnosis of T1D for at least one year with positivity for at least one diabetes-associated antibody (GADA, ZnT8A, IAA, or IA-2 A); Caucasian ethnicity (the most prevalent ethnicity among children and adolescents with T1D followed up in our center). Exclusion criteria: chronic diseases other than T1D, tubular or glomerular pathologies, cardiovascular diseases, or other metabolic diseases. The local Institutional Ethics Committee approved the study protocol (protocol number 25212, April 26, 2021), and written informed consent was obtained from all study participants aged 6 years and above, as well as their parents, prior to enrollment.

Anthropometric measurements [height, body weight, waist circumference (WC)], and blood pressure (BP) were collected. The waist circumference is expressed as the average of at least two or three measurements. The BMI Z-scores were calculated using age- and sex-specific BMI percentiles based on the WHO child growth standards [23]. Body composition parameters, including fat mass (kg), fat-free mass (kg), and fat mass percentage (FM%), were assessed using bioelectrical impedance analysis (BIA) with a Tanita PRO AM-180. Fat Mass Index (FMI) was calculated by dividing fat mass (kg) by height (m) squared. Waist-to-height ratio (WHtR) was calculated by dividing WC by height; both WC and height were expressed in centimeters.

Pubertal status (Tanner stages I-V) [24], age at onset, duration of T1D, insulin treatment modalities (MDI, IP, and AID systems), total daily doses (TDD) of insulin per kilogram of body weight (U/kg), and glucose metrics were recorded for all participants. In line with international recommendations, the following CGM metrics were calculated and expressed as time percentage: time in range 70–180 mg/dL (3.9–10.0 mmol/L) (TIR); time below range < 70 mg/dL (< 3.9 mmol/L) (TBR); time below range 55–69 mg/dL (3.0-3.9 mmol/L) (low glucose or Level 1 hypoglycemia) (TBR1); time below range < 54 mg/dL (3.0 mmol/L) (very low glucose or Level 2 hypoglycemia) (TBR2); time above range 181–250 mg/dL (10.1–13.9 mmol/L) (high glucose or Level 1 hyperglycemia) (TAR1); time above range > 250 mg/dL (> 13.9 mmol/L) (very high glucose or Level 2 hyperglycemia) (TAR2). Glycemia Risk Index (GRI) was calculated as previously described [25].

### Biochemical parameters

Venous blood samples were collected from all participants during the enrollment visit, after they signed the informed consent form and verified their inclusion criteria. Hemoglobin A1c was measured using an automated cation-exchange high-performance liquid chromatography (Bio-Rad), calibrated following the approved standards of the Diabetes Control and Complications Trial (DCCT). All other biochemical parameters [i.e., triglycerides (TG), total cholesterol, high-density lipoprotein (HDL) cholesterol, and low-density lipoprotein cholesterol] were analyzed in a single reference centralized laboratory according to International Standards. Low-density lipoprotein (LDL) cholesterol was calculated using Friedewald’s equation.

### Statistical analysis

Data were analyzed using the SPSS v26.0 software package (SPSS Inc., Chicago, USA). The Kolmogorov-Smirnov test was used to assess the normality of the distributions. Data are presented as means and standard deviations or medians and interquartile ranges (IQR) as appropriate. For categorical variables, absolute values and relative frequencies are presented. Unpaired Student’s *t*-test, Mann-Whitney test, or chi-square test were used where appropriate to compare data between subjects of different sexes. ANOVA was performed to compare data between subgroups defined by the type of treatment. Post hoc Bonferroni analyses were performed to assess pairwise differences between these subgroups.

The relationships between age, diabetes duration, BMI Z-score, FMI, WHtR, TDD, HbA1c, and CGM metrics were evaluated using Spearman’s correlation coefficient. A post-hoc evaluation of our sample size revealed that our cohort (*n* = 364; males, *n* = 194; females, *n* = 170) has more than adequate statistical power to detect the observed correlations. In fact, Spearman coefficients in the range of 0.18–0.25—several of which were even higher—correspond to a statistical power exceeding 85–95% at α = 0.05 in both sexes. This confirms that the significant associations reported in the correlation analyses are unlikely to be affected by type II error and reflect robust relationships within the study population.

Finally, logistic regression models were performed to assess the main independent predictors of HbA1c < 7.0% and TIR > 70%. FMI, WHtR, TDD, and treatment modalities were included as independent variables, along with sex, age, diabetes duration, and pubertal stage.

## Results

### Physical, anthropometric, biochemical characteristics and glycemic control metrics: comparison by sex

The anthropometric characteristics and body composition of the study cohort, comprising 364 children and adolescents with T1D, are stratified by sex (males, 53.3%) and reported in Table 1. Age, diabetes duration, BMI, BMI Z-score, WC, WHtR, DBP, and TDD were not statistically different between males and females. Fat mass and FMI were significantly (*p* < 0.001) higher in females than in males, whereas fat-free mass and SBP were significantly higher (*p* < 0.001 and *p* < 0.05, respectively) in males than in females. More females were in the pubertal or post-pubertal stage than males (*p* < 0.01).

**Table 1:** Clinical, demographic, and biochemical characteristics of children and adolescents with T1D, stratified by sex

	Male (*n* = 194)	Female (*n* = 170)	Total (*n* = 364)	*p*-value
Age (years)	14.2 [11.5–17.1]	14.7 [11.7–17.2]	14.4 [11.5–17.1]	0.991
Diabetes duration (years)	6.6 [3.3–10.2]	7.2 [4.3–10.5]	6.9 [4.1–10.3]	0.253
*Puberty*				
Prepubertal n (%)	45 (23.2)	28 (16.5)	73 (20.1)	
Pubertal n (%)	58 (29.9)	37 (21.8)	95 (26.1)	
Postpubertal n (%)	91 (46.9)	105 (61.8)	196 (53.8)	
Height (cm)	**166.7 [150.9–175.0]**	**160.0 [152.0–165.0]**	**162.0 [151.0–169.0]**	**< 0.001**
BMI (kg x m^− 2^)	**20.3 [18.0–23.0]**	**21.4 [19.2–23.8]**	**20.7 [18.5–23.5]**	0.067
BMI [kg x (m^− 2^)] Z-score	*0.48 [-0.33–1.30]*	*0.66 [0.09–1.20]*	*0.58 [-0.11–1.22]*	*0.102*
WC (cm)	75.0 [67.7–79.0]	72.0 [66.0–78.0]	73.0 [66.0–79.0]	0.063
WHtR	0.45 [0.42–0.48]	0.46 [0.42–0.50]	0.46 [0.42–0.49]	0.866
Fat Mass (%)	**19.3** ± **6.2**	**27.3** ± **5.6**	**23.0** ± **7.0**	**< 0.001**
Fat Mass (kg)	**9.0 [6.7–13.4]**	**14.5 [10.1–19.1]**	**11.6 [7.7–16.3]**	**< 0.001**
Free Fat Mass (kg)	**47.2 [33.0–55.5]**	**40.1 [33.1–45.8]**	**42.9 [33.0–51.1]**	**< 0.001**
Fat Mass Index (kg x m^− 2^)	**3.5 [2.7–5.3]**	**5.6 [4.4–7.3]**	**4.6 [3.3–6.2]**	**< 0.001**
SBP (mm Hg)	108.4 ± 9.8	106.2 ± 9.8	107.3 ± 9.9	**0.025**
DBP (mm Hg)	68.7 ± 6.7	68.6 ± 7.3	68.6 ± 7.0	0.874
Total cholesterol (mg x dL^− 1^)	154.5 ± 25.1	165.0 ± 28.6	159.3 ± 27.3	**0.001**
HDL cholesterol (mg x dL^− 1^)	63.0 ± 14.3	64.0 ± 14.2	63.5 ± 14.3	0.457
LDL cholesterol (mg x dL^− 1^)	79.2 ± 21.9	87.7 ± 22.5	83.1 ± 22.5	< **0.001**
TG (mg x dL^− 1^)	51.0 [40.7–62.0]	55.0 [42.2–70.6]	53.0 [42.0–66.0]	**0.046**
*Type of Treatment*				
MDI n (%)	94 (48.5)	85 (50.0)	179 (49.2)	0.909
CSII n (%)	52 (26.8)	42 (24.7)	94 (25.8)	0.909
AID n (%)	48 (24.7)	43 (25.3)	91 (25.0)	0.909
HbA1c (%)	**7.5** ± **0.9**	**7.8** ± **1.1**	**7.6** ± **1.0**	**0.016**
TBR 2 (%)	0.0 [0.0–1.0]	0.0 [0.0–1.0]	0.0 [0.0–1.0]	0.909
TBR 1 (%)	2.0 [1.0–4.0]	2.0 [1.0–3.0]	2.0 [1.0–4.0]	0.287
TIR (%)	56.0 ± 14.8	54.4 ± 16.5	55.2 ± 15.6	0.327
TAR 1 (%)	24.9 ± 6.5	26.3 ± 7.8	25.6 ± 7.2	**0.044**
TAR 2 (%)	13.0 [6.0–24.0]	13.0 [6.7–24]	13.0 [6.0–24.0]	0.764
CV (%)	38.4 ± 5.8 / 38.0 [34.0–42.0]	36.9 ± 5.0 / 36.0 [33.0–40.0]	37.7 ± 5.5 / 37.0 [34.0–41.0]	**0.011**
GRI	49.2 [36.0–70.2]	52.8 [39.1–70.0]	51.2 [37.4–70.0]	0.560
TDD (U x kg ^− 1^)	0.8 ± 0.2	0.8 ± 0.2	0.8 ± 0.2	0.728

Sample size, *n* = 364, except where indicated. Data are presented as mean ± SD, median [IQR], or number and percentages as appropriate

BMI, Body mass index; WC, Waist circumference; WHtR, Waist circumference to height ratio; SBP, Systolic blood pressure; DBP, Diastolic blood pressure; HDL, High-density lipoprotein; LDL, Low-density lipoprotein; TG, Triglycerides; TBR2, Time below range < 54 mg/dL (< 3.0 mmol/L) (very low glucose or Level 2 hypoglycemia); TBR1, Time below range 54–69 mg/dL (3.0–3.9 mmol/L) (low glucose or Level 1 hypoglycemia); TIR, Time in range 70–180 mg/dL (3.9–10.0 mmol/L);; TIR, Time in range; TAR1, Time above range 181–250 mg/dL (10.1–13.9 mmol/L) (high glucose or Level 1 hyperglycemia); TAR2, Time above range > 250 mg/dL (> 13.9 mmol/L) (very high glucose or Level 2 hyperglycemia); CV, Coefficient of variation; GRI, Glycaemic risk index; TDD, Total daily dose of insulin. CGM metrics (i.e., TBR, TIR, TAR) were assessed in a time interval of 90 days

Fields in bold indicate significant differences as result of the *t*-Student test or Mann-Whitney U-Test (*p* ≤ 0.05)

Females had significantly higher values of total cholesterol (*p* < 0.001), LDL cholesterol (*p* < 0.001), and TG (*p* < 0.05) than males. HDL-cholesterol values were not significantly different between sexes. Females had significantly higher (*p* < 0.05) levels of HbA1c and TAR1 than males. No significant differences between males and females were found in TBR 2, TBR 1, TIR, TAR2, CV, and GRI. The TDD and treatment modalities, i.e., MDI, IP, or AID system, were not significantly different between the sexes.

### Physical, anthropometric, biochemical characteristics, and glycemic control metrics: comparison by treatment modalities

Children and adolescents with T1D using MDI were significantly older (*p* < 0.05) than those using IP or AID systems (Table 2). Children and adolescents using AID systems had significantly lower WC (*p* < 0.001) and fat-free mass (*p* < 0.05) than those using MDI or IP. Fat mass, FM%, FMI, and WHtR were not significantly different among the three groups, as well as blood pressure, total cholesterol, HDL-cholesterol, and LDL-cholesterol. Children and adolescents with T1D using the AID system had significantly lower levels of TG (*p* < 0.02), HbA1c (*p* < 0.001), TAR1 (*p* < 0.02), TBR2 (*p* < 0.04), and TBR1 (*p* < 0.03) than the two other groups. Children and adolescents with T1D using the AID system or IP had significantly (*p* < 0.001) higher TIR and lower CV than those using MDI. GRI was significantly (*p* < 0.01) lower in those using IP than in the two other groups. The TDD was significantly (*p* < 0.01) higher in children and adolescents with T1D using MDI than in the two comparison groups.

**Table 2:** Clinical, demographic, and biochemical characteristics of children and adolescents with T1D, stratified by type of treatment

	MDI (*n* = 179)	*IP* (*n* = 94)	AID (*n* = 91)	*p*-value
Age (years)	15.2 [11.9–17.2]	14.2 [11.7–17.1]	14.0 [10.0–16.6]	0.051
Sex, M/F (n)	94 / 85	52 / 42	48 / 43	0.901
Diabetes duration (years)	**6.5 [3.1–10.2]**	**7.7 [5.0–10.1]**	**7.2 [4.3–10.6]**	**0.039**
Puberty				
Prepubertal n (%)	27 (15.1)	18 (19.1)	28 (30.8)	
Pubertal n (%)	50 (27.9)	27 (28.7)	18 (19.8)	**0.039**
Postpubertal n (%)	102 (57.0)	49 (52.1)	45 (49.5)	
Height (cm)	**163.0 [154.5–170.0] c**	**162.8 [153.7–170.2]**	**159.0 [142.7–167.5] c**	**0.017**
BMI (kg x m^− 2^)	21.0 [19.1–23.8]	20.2 [18.5–23.0]	20.2 [17.5–22.3]	0.08
BMI [kg x (m^− 2^)] Z-score	0.65 [-0.05–1.23]	0.60 [-0.33–1.22]	0.49 [-0.12–1.19]	0.626
WC (cm) (*n* = 314)	**75.0 [68.0–80.0] d**	**72.0 [65.5–78.5]**	**70.0 [65.0–75.5] d**	**0.006**
WHtR (*n* = 314)	0.46 [0.43–0.49]	0.45 [0.42–0.48]	0.44 [0.41–0.50]	0.191
Fat Mass (%)	23.5 ± 7.3	22.0 ± 6.2	22.6 ± 6.9	0.225
Fat Mass (kg)	12.3 [8.2–16.9]	11.0 [7.7–16.0]	10.1 [6.8–15.5]	0.061
Free Fat Mass (kg)	**44.1 [33.6–52.6] c**	**44.0 [34.5–51.6] a**	**39.5 [27.4–48.0] c**	**0.034**
Fat Mass Index (kg x m^− 2^)	4.8 [3.3–6.4]	4.6 [3.2–5.8]	4.2 [3.2–6.1]	0.208
SBP (mm Hg) (*n* = 348)	107.9 ± 9.2	107.4 ± 9.3	105.8 ± 11.5	0.191
DBP (mm Hg) (*n* = 348)	69.1 ± 6.8	68.6 ± 7.0	67.6 ± 7.4	0.353
Total cholesterol (mg x dL^− 1^)	160.4 ± 27.5	157.4 ± 28.6	159.2 ± 23.1	0.860
HDL cholesterol (mg x dL^− 1^)	62.2 ± 14.1	64.7 ± 13.6	64.8 ± 15.4	0.275
LDL cholesterol (mg x dL^− 1^)	84.2 ± 23.1	81.1 ± 24.4	82.8 ± 19.2	0.524
TG (mg x dL^− 1^)	**55.0 [44.0–70.0] c**	**51.0 [41.5–61.5]**	**48.0 [39.0–64.0] c**	**0.016**
HbA1c (%) (*n* = 350)	**7.9 ± 1.1 b**,** d**	**7.4 ± 0.7 b**	**7.2 ± 0.9 d**	**< 0.001**
TBR 2 (%)	**0.0 [0.0–1.0] c**	**0.0 [0.0–1.0]**	**0.0 [0.0–1.0] c**	**0.034**
TBR 1 (%)	**2.0 [1.0–4.0] c**	**2.0 [1.0–3.2]**	**1.0 [1.0–3.0] c**	**0.027**
TIR (%)	**49.1 ± 15.6 b**,** d**	**60.5 ± 12.7 b**	**61.8 ± 13.6 d**	**< 0.001**
TAR 1 (%)	**26.7 ± 6.8 c**	**24.9 ± 7.2 a**	**24.3 ± 7.7 c**	**0.019**
TAR 2 (%)	**20.0 [9.0–30.0] b**,** d**	**9.0 [5.0–15.0] b**	**9.5 [5.7–15.5] d**	**< 0.001**
CV (%)	**39.1 ± 5.9 / 39.0 [34.8–42.5] b**,** d**	**36.3 ± 4.5 / 36.0 [33.0–39.0] b**	**36.3 ± 5.0 / 35.0 [32.5–40.2] d**	**< 0.001**
GRI	**54.4 [39.6–75.4] b**	**45.8 [34.0–57.0] b**	**51.6 [37.9–67.2]**	**0.003**
TDD (U x kg ^− 1^)	**0.9 ± 0.2 b**,** c**	**0.8 ± 0.2 b**	**0.8 ± 0.2 c**	**0.003**

Sample size, *n* = 364, except where indicated. Data are presented as mean ± SD, median [IQR], or number and percentages as appropriate

MDI, Multiple daily injections; CSII, Continuous subcutaneous insulin infusion; AID, Automated insulin delivery; BMI, Body mass index; WC, Waist circumference; WHtR, Waist circumference to height ratio; SBP, Systolic blood pressure; DBP, Diastolic blood pressure; HDL, High-density lipoprotein; LDL, Low-density lipoprotein; TG, Triglycerides; TBR2, Time below range < 54 mg/dL (< 3.0 mmol/L) (very low glucose or Level 2 hypoglycemia); TBR1, Time below range 54–69 mg/dL (3.0–3.9 mmol/L) (low glucose or Level 1 hypoglycemia); TIR, Time in range 70–180 mg/dL (3.9–10.0 mmol/L);; TIR, Time in range; TAR1, Time above range 181–250 mg/dL (10.1–13.9 mmol/L) (high glucose or Level 1 hyperglycemia); TAR2, Time above range > 250 mg/dL (> 13.9 mmol/L) (very high glucose or Level 2 hyperglycemia); CV, Coefficient of variation; GRI, Glycaemic risk index; TDD, Total daily dose of insulin. CGM metrics (i.e., TBR, TIR, TAR) were assessed in a time interval of 90 days

Fields in bold indicate significant differences as result of One Way ANOVA or Kruskal Wallis test (*p* ≤ 0.05)

Pairwise analysis was performed and statistically significant differences (Bonferroni adj-*p* ≤ 0.05) as represented by lower case letters as follow:

MDI versus CSII: a < 0.05; b < 0.01;

MDI versus AID: c < 0.05; d < 0.01;

CSII versus AID: e < 0.05; f < 0.01

### Correlation analyses

Spearman correlations between HbA1c, CGM metrics, age, diabetes duration, TDD, and adiposity measures are shown in Supplementary Table 1.

In males, the HbA1c was significantly correlated with T1D duration (rho = 0.22, *p* < 0.01) and FMI (rho = 0.19, *p* < 0.05), whereas in females, HbA1c was significantly correlated with age (rho = 0.19, *p* < 0.05), FM% (rho = 0.19, *p* < 0.01), FMI (rho = 0.24, *p* < 0.01), and WHtR (rho = 0.20, *p* < 0.05). In males, the TIR was significantly correlated with diabetes duration (rho = -0.23, *p* < 0.01), BMI Z-score (rho = -0.16, *p* < 0.01), and FMI (rho = -0.185, *p* < 0.01), whereas in females, it was correlated with FM% (rho = -0.22, *p* < 0.01), FMI (rho = -0.19, *p* < 0.05), and WHtR (rho = -0.24, *p* < 0.01). In males, TBR2 was significantly correlated with diabetes duration (rho: 0.15, *p* < 0.05), age (rho = 0.18, *p* < 0.05), BMI Z-score (rho = 0.18, *p* < 0.05), and FMI (rho = 0.17, *p* < 0.05), whereas no significant correlations were found in females. In both sexes, the TBR1 did not significantly correlate with age, diabetes duration, or adiposity measures.

In females, the TAR1 was significantly correlated with age (rho = 0.17, *p* < 0.05) and FMI (rho = 0.16, *p* < 0.05), whereas no significant correlations were found in males. In males, TAR2 was significantly associated with diabetes duration (rho: 0.19, *p* < 0.01), and in females with FM% (rho = 0.19, *p* < 0.05). In males, the CV is positively correlated with age (*p* < 0.01), diabetes duration, and all adiposity measures. In females, it is positively correlated with the WHtR (rho = 0.21, *p* < 0.05).

In females, the total daily dose (TDD) was significantly correlated with age (rho = -0.16, *p* < 0.05) and FMI (rho = 0.252, *p* < 0.01). In males, TDD showed a significant positive correlation with BMI Z-score (rho = 0.21, *p* < 0.01). No other significant correlations were observed between TDD and diabetes duration or adiposity measures.

### Logistic regression analyses

**Fig. 1:**
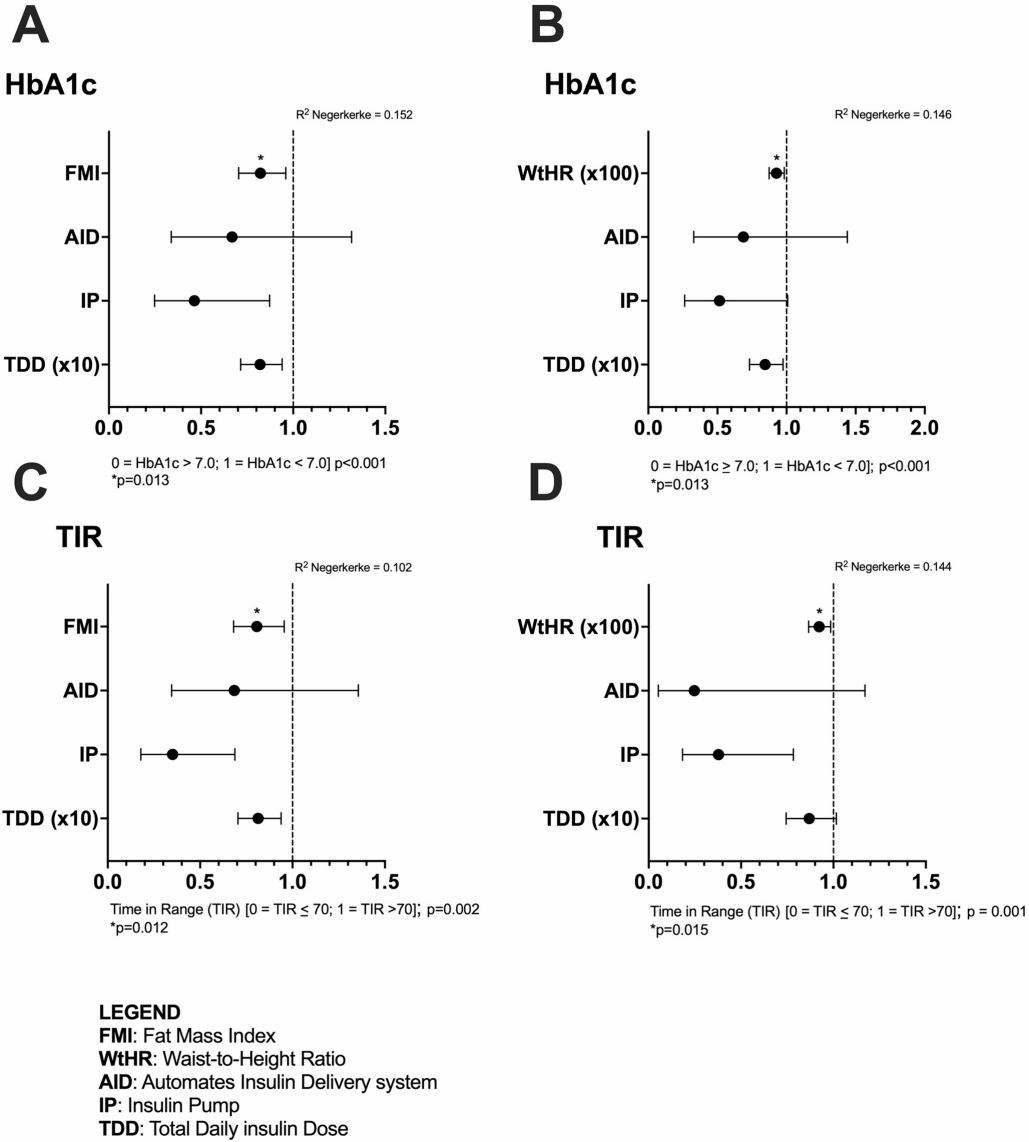
Forest plot of the logistic regression analysis. HbA1c < 7% and TIR > 70% were significantly predicted by FMI and WHtR, independently of TDD, treatment modalities, and the other independent variables (sex, age, diabetes duration, and puberty). Panel **A**: HbA1c < 7% was significantly predicted by FMI; Panel **B**: HbA1c < 7% was significantly predicted by WHtR; Panel C: TIR > 70% was significantly predicted by FMI; Panel D: TIR > 70% was significantly predicted by WHtR

The main results of the logistic regression models, which used the main glycemic control parameters (HbA1c and TIR) as dependent variables and FMI, WHtR, TDD, and treatment modalities, sex, age, diabetes duration, and puberty as independent variables, are presented in Table 3. HbA1c < 7% and TIR > 70% were significantly predicted by FMI [OR (95%CI): 0.822 (0.704–0.960), *p* = 0.013, and 0.807 (0.681–0.955), *p* = 0.012, respectively) and WHtR(*100) [OR (95%CI): 0.927 (0.874–0.983), *p* = 0.013, and 0.923 (0.866–0.985), *p* = 0.015, respectively], independently of TDD, treatment modalities, and the other independent variables (Fig. 1).

**Table 3:** Four models of binary logistic regression

Dependent variable	Covariates in logistic regression models*	OR (95% CI)	*P*-value	Dependent variable	Covariates in logistic regression models*	OR (95% CI)	*P*-value
[0 = HbA1c ≥ 7.0; 1 = HbA1c < 7.0] *p* < 0.001 R^2^ Negerkerke = 0.152	TDD (x10)	0.820 (0.715–0.940)	**0.004**	[0 = HbA1c ≥ 7.0; 1 = HbA1c < 7.0] *p* < 0.001 R^2^ Negerkerke = 0.146	TDD (x10)	0.845 (0.732–0.975)	**0.021**
	Treatment modality- MDI		0.058		Treatment modality- MDI		0.153
	Treatment modality- IP	0.464 (0.248–0.872)	**0.017**		Treatment modality- IP	0.515 (0.263–1.008)	0.515
	Treatment modality-AID	0.668 (0.338–1.317)	0.244		Treatment modality-AID	0.688 (0.329–1.440)	0.321
	FMI	0.822 (0.704–0.960)	**0.013**		WHtR (x 100)	0.927 (0.874–0.983)	**0.013**
Time in Range (TIR) [0 = TIR≤70; 1 = TIR > 70] *p* = 0.002 R^2^ Negerkerke = 0.102	TDD (x 10)	0.814 (0.705–0.938)	**0.005**	Time in Range (TIR) [0 = TIR≤70; 1 = TIR > 70] *p* = 0.001 R^2^ Negerkerke = 0.144	TDD (x10)	0.869 (0.744–1.016)	0.0781
	Treatment modality- MDI		**0.008**		Treatment modality- MDI		**0.030**
	Treatment modality- IP	0.351 (0.179–0.688)	**0.002**		Treatment modality- IP	0.378(0.183–0.783)	**0.009**
	Treatment modality-AID	0.685 (0.346–1.356)	0.277		Treatment modality-AID	0.247 (0.052–1.171)	0.367
	FMI	0.807 (0.681–0.955)	**0.012**		WHtR (x100)	0.923 (0.866–0.985)	**0.015**

The bold is for the significant differences between variables

Dependent variables: HbA1c and TIR. Independent variables: total daily insulin dose (TDD), treatment modalities, FMI (left) or WHtR (right)

*The following variables were also included in the models: sex, age, diabetes duration, and pubertal stage; none of these significantly predicted the dependent variables

## Discussion

The results of this study confirmed the hypothesis that body adiposity affects glycemic control in children and adolescents with T1D, regardless of the treatment modality used (MDI, IP, or AID).

Data from the T1D registries showed that the prevalence of overweight and obesity in children and adolescents has significantly increased in the last decades [13, 15, 26]. This trend is consistent with the findings reported for the general population worldwide [12]. Several lifestyle and environmental factors contribute to this trend, reflecting environmental changes that may exacerbate the negative impact of obesity-related comorbidities [27, 28].

Nonetheless, what is recommended in the international guidelines may not be sufficient to prevent excess body fat accumulation [2]. Moreover, despite the recent implementation of technological devices, several pieces of evidence have demonstrated that achieving optimal management of T1D and maintaining good glycemic control without significant weight gain can be challenging [29]. Indeed, although HbA1c and glucose metrics can be improved by using IP and AID systems, a greater increase in BMI has been reported in children and adolescents who use these devices compared to those who do not [20, 22].

Longitudinal data showed that adolescents with a higher BMI were significantly less likely to achieve international glycemic targets [21]. Moreover, increasing evidence has demonstrated that adiposity per se, rather than BMI, plays a critical role in the exposure to CVRFs and hypertension [19]. A systematic review and meta-analysis also reported that children and adolescents with T1D tend to have approximately 9% higher body fat than their healthy peers [30].

In this study, we showed that as fat mass increases, the quality of glycemic control deteriorates. In fact, as shown by the logistic regression analysis, the probability of achieving *at-target* glycemic control (HbA1c < 7.0% and TIR > 70%) increases by ~ 19% per one-unit increase in FMI or approximately 7% per one-unit increase in WHtR (*100). Although the magnitude of the impact of adiposity, adjusted for covariates, is relatively modest, these findings align with previous studies, showing that increased fat mass, particularly visceral adiposity, impairs glycemic control by promoting increased insulin requirements [30]. In addition, the results of this study further emphasize that fat mass and increased adiposity are key factors associated with a higher prevalence of diabetes-related complications and may compromise long-term health outcomes [31, 32].

A sex-based difference in life expectancy was also reported by several studies, with females with T1D exhibiting a lower life expectancy compared to their male peers [33]. Interestingly, the DCCT/Epidemiology of Diabetes Interventions and Complications (DCCT/EDIC) study showed that although women with T1D had more favorable cardiometabolic risk factor profiles than men, there was no corresponding benefit in CVD risk [34]. Furthermore, females have less favorable glycemic control and higher fat mass than males from adolescence onward [35]. Our study confirms these findings, reporting higher body adiposity and a stronger negative association between adiposity and glycemic control in females than males. Additionally, WHtR, i.e., a gross index of abdominal body fat that promotes metabolic dysregulation and increases exposure to CVRFs, was more negatively associated with glycemic control in females compared to males [36, 37]. Nevertheless, logistic regression analysis showed a significant relationship between body fat or WHtR and glycemic control, independent of sex, suggesting that total adiposity or abdominal fat accumulation, per se, are essential in both girls and boys.

In our study, children and adolescents treated with AID systems reported better glycemic control than those using MDI or IP, as previously reported [8]. However, the fat mass was not significantly different in children and adolescents using MDI, IPs, or AID systems, suggesting that adiposity plays a role in glycemic control, independent of the treatment modalities used. Logistic regression analyses confirmed this finding as FMI predicted HbA1c < 7% and TIR > 70%, independent of sex, age, puberty, diabetes duration, and treatment modalities. Analogous results have been obtained using the WHtR instead of FMI. The WHtR is associated with cardiovascular and metabolic risk factors in children and adolescents; however, unlike other obesity indices, it is easy to measure, inexpensive, and does not require reference tables, making it straightforward to use in clinical practice [38].

The results of this study highlight the importance of addressing body adiposity in children and adolescents with T1D. The consequences of overweight and obesity on glycemic control are more significant for children and adolescents with T1D than for the general population, thus suggesting the need to prevent and treat overweight and obesity as soon as possible. Indeed, recent studies focusing on childhood and adolescent obesity have reported better clinical outcomes when interventions commence early [39]. Our results showed that adiposity reduces the positive impact of advanced technology on diabetes care in individuals with T1D. Therefore, the clinicians’ efforts should be directed to the optimal use of pumps or AID systems and in acquiring and maintaining adequate control of body composition. Nutrition, physical activity, and lifestyle remain paramount in diabetes care, and their role cannot be overlooked or neglected. Appropriate support to children, adolescents, and their families is necessary to improve these three critical factors affecting metabolic regulation, as suggested by the ADA and ISPAD recommendations [1, 2].

Some limitations should be considered when interpreting the results of this study: (i) Given the cross-sectional and exploratory design of this study, the directionality of the association between adiposity and glycemic control cannot be determined. Increased adiposity may worsen metabolic control through greater insulin resistance and higher insulin requirements, whereas suboptimal glycemic control may also contribute to fat mass gain through intensified insulin therapy, reduced glycosuria, and behavioral factors. Longitudinal studies are needed to clarify this bidirectional relationship; (ii) all the recruited children and adolescents were with European ancestry, therefore, these findings cannot be generalized to individuals with T1D from other ethnic backgrounds; (iii) data on physical activity, dietary habits and socioeconomic status, which represent important potential confounders, were not collected in this cohort; (iiii) body composition measured by BIA, although DXA would provide more accurate measurements. However, DXA has some limitations, including X-ray exposure and higher costs. Moreover, the WHtR has been reported to be the most accurate anthropometric index of visceral fat mass, as measured by DXA, in individuals with T1D [40].

This study has several strengths: (i) it includes a relatively large, real-world cohort of children and adolescents with T1D recruited from a single centre; (ii) anthropometric, BIA, derived body composition parameters, biochemical, and glycemic data were collected by the same operators, ensuring consistency and reducing measurement variability; (iii) the study involved participants using the three main treatment modalities currently available - MDI, IP and AID; (iv) glycemic control was assessed through a comprehensive set of CGM metrics; (v) although the proportion of participants using insulin pumps or AID systems reflects current practice patterns in Italy, where access remains more limited than in other countries, this characteristic offers valuable insight into metabolic outcomes under real-world technological constraints and helps contextualize the generalizability of our findings. Overall, to our knowledge, this is the first study to examine fat mass and fat distribution in relation to glycemic outcomes across all treatment modalities within an Italian pediatric cohort—an element that adds both novelty and clinical relevance, especially given the growing recognition of FMI and WHtR as meaningful markers of cardiometabolic risk in youth.

In conclusion, the findings of this study suggest that fat mass is significantly associated with glycemic control in children and adolescents with T1D, independently of the treatment modalities. These results highlight the importance of maintaining adiposity within physiological ranges to support optimal metabolic control and improve long-term diabetes management in youth. Future studies are needed to better understand the underlying mechanisms linking adiposity and glycemic control in children and adolescents with T1D, and to evaluate the effectiveness of targeted interventions in *optimizing* body composition in this population.

## Supplementary Information

Below is the link to the electronic supplementary material.

Supplementary Material 1

Supplementary Material 2

## Abbreviations

ADA: American Diabetes Association
AID: Automated insulin delivery
BMI: Body mass index
BP: blood pressure
CGM: Continuous glucose monitoring
CVRFs: Cardiovascular risk factors
FM: Fat mass
FMI: Fat mass index
GRI: Glycemia risk index
HDL: High-density lipoprotein cholesterol
IP: Insulin Pump
ISPAD: International Society for Pediatric and Adolescent Diabetes
LDL: Low-density lipoprotein
MDI: Multiple daily injection
PwD: People with type 1 diabetes
TAR: Time above range
TBR: Time below range
TDD: Total daily dose/kg of body weight
TG: Triglycerides (TG)
TIR: Time in range
T1D: Type 1 diabetes
WC: Waist circumference
WHtR: Waist-to-height ratio
