# Supplementary material for: Relationship between body adiposity and glycemic control in children and adolescents with type 1 diabetes
Source: Acta Diabetol. 2026 Jan 31;63(4):665–75. doi: 10.1007/s00592-026-02643-2 (PMC13092533; doi:10.1007/s00592-026-02643-2)
Supplement: Supplementary file 1 — Supplementary Material 1 [file 592_2026_2643_MOESM1_ESM.docx]

**Supplementary Table 1**. Spearman bivariate correlation between HbA1c, glycemic metrics, TDD, diabetes duration, age, BMI z-score, fat mass index, fat mass percentage, and WHtR.

|  | **Age** | | **Diabetes duration (years)** | | **BMI Z-score** | | **Fat Mass Index (FMI)** | | **WHtR** | |
| --- | --- | --- | --- | --- | --- | --- | --- | --- | --- | --- |
|  | **Male** | **Female** | **Male** | **Female** | **Male** | **Female** | **Male** | **Female** | **Male** | **Female** |
| HbA1c (%) | 0.102 | **0.192*** | **0.219^**^** | 0.070 | 0.116 | 0.076 | **0.186^*^** | **0.238**** | 0.046 | **0.198*** |
| TBR2 | **0.180^*^** | 0.008 | **0.148^*^** | 0.023 | **0.182^*^** | 0.001 | **0.174^*^** | 0.015 | 0.078 | 0.125 |
| TBR1 | 0.113 | -0.007 | -0.013 | 0.029 | 0.087 | 0.112 | 0.080 | 0.071 | 0.076 | 0.137 |
| TIR | -0.015 | -0.097 | **-0.228^**^** | 0.067 | **-0.160^*^** | -0.108 | **-0.185**** | **-0.190*** | -0.076 | **-0.242**** |
| TAR1 | -0.005 | **0.174*** | 0.136 | -0.049 | 0.051 | 0.059 | 0.126 | **0.163*** | 0.055 | 0.058 |
| TAR2 | -0.032 | 0.067 | **0.192^**^** | -0.057 | 0.132 | 0.058 | 0.132 | 0.142 | 0.053 | **0.212*** |
| CV (%) | **0.575**** | -0.052 | **0.255**** | -0.032 | **0.482**** | 0.075 | **0.470**** | 0.066 | **0.546**** | **0.212*** |
| TDD (U x kg^-1^) | 0.081 | **-0.16*** | 0.123 | -0.50 | **0.21**** | 0.09 | 0.12 | **0.252**** | 0.05 | 0.042 |

* p < 0.05, ** p < 0.01

Abbreviations: BMI, Body mass index; TBR2, Time below range <54 mg/dL (<3.0 mmol/L) (very low glucose or Level 2 hypoglycemia); TBR1, Time below range 54-69 mg/dL (3.0 - 3.9 mmol/L) (low glucose or Level 1 hypoglycemia); TIR, Time in range 70–180 mg/dL (3.9–10.0 mmol/L); TAR1, Time above range 181-250 mg/dL (10.1-13.9 mmol/L) (high glucose or Level 1 hyperglycemia); TAR2, Time above range >250 mg/dL (>13.9 mmol/L) (very high glucose or Level 2 hyperglycemia); GRI, Glycaemic risk index; TDD, Total daily insulin dose.
