# Supplementary material for: Relationship between body adiposity and glycemic control in children and adolescents with type 1 diabetes
Source: Acta Diabetol. 2026 Jan 31;63(4):665–75. doi: 10.1007/s00592-026-02643-2 (PMC13092533; doi:10.1007/s00592-026-02643-2)
Supplement: Supplementary file 2 — Supplementary Material 2 [file 592_2026_2643_MOESM2_ESM.pdf]

Springer

Conflict of Interest Disclosure Form

It is the policy of the Journal *Acta Diabetologica* to ensure balance, independence, objectivity, and scientific rigor in the Journal. All authors are expected to disclose to the readers any real or apparent conflict(s) of interest that may have a direct bearing on the subject matter of the article. This pertains to relationships with pharmaceutical companies, biomedical device manufacturers or other corporations whose products or services may be related to the subject matter of the article or who have sponsored the study.

The intent of the policy is not to prevent authors with a potential conflict of interest from publication. It is merely intended that any potential conflict should be identified openly so that the readers may form their own judgements about the article with the full disclosure of the facts. It is for the readers to determine whether the authors' outside interest may reflect a possible bias in either the exposition of the conclusions presented.

The corresponding author will complete and submit this form to the Editor-in-Chief on behalf of all authors listed below.

Article Title ....

Relationship between Body Adiposity and Glycemic Control in Children and Adolescents with Type 1 Diabetes

Authors

Claudio Maffeis, Ilaria Fierri, Elisa Morotti, Erika Caiazza, Quincy Pedranzini, Marco Marigliano, Claudia Piona

I certify that there is no actual or potential conflict of interest in relation to this article. If any conflict exists, please define hereafter:

Conflict (if none, "None" or describe financial interest/arrangement with one or more organizations that could be perceived as a real or apparent conflict of interest in the context of the subject of this article):

CM has received research funding from Medtronic, Sanofi, and Movi, has received honoraria for lectures from Abbott, Sanofi, and Eli Lilly, has been supported for attending meetings from Sanofi, and has participated in Data Monitoring for Abbott and Sanofi. MM has received honoraria for lectures from Theras, Novo Nordisk, Ypsomed, and Medtronic, has been supported for attending meetings from Movi and Abbott, and has participated in Data Monitoring for Movi. CP has received honoraria for lectures from Sanofi and has been endorsed for attending meetings from Movi and Abbott. IF, EM, EC, and QP have no potential conflict of interest to declare.

Name Marco Marigliano

Signature .... Date 20/10/2025

Please also insert a "conflict of interest statement" in the manuscript itself.

Please upload this form together with your manuscript at online submission.
